# Practice Trends of Fibrinogen Monitoring in Thrombolysis

**DOI:** 10.3390/jcm7050111

**Published:** 2018-05-10

**Authors:** Claire Kaufman, Thomas Kinney, Keith Quencer

**Affiliations:** 1 Department of Radiology, University of Utah, Salt Lake City, UT 84132, USA; keith.quencer@hsc.utah.edu; 2 Department of Radiology, University of California San Diego, San Diego, CA 92103, USA; tbkinney@ucsd.edu

**Keywords:** thrombolysis, fibrinogen, deep venous thrombosis, tissue plasminogen activator

## Abstract

There is a lack of evidence or societal guidelines regarding the utility of fibrinogen monitoring during thrombolysis. The purpose of our study was to investigate the current use of monitoring fibrinogen levels during thrombolysis. A voluntary, anonymous online survey was sent to all physician members of the Society of Interventional Radiology, consisting of 23 questions related to practitioner demographics, thrombolysis protocol, and fibrinogen monitoring. There were 455 physician responses; 82% of respondents monitored fibrinogen levels during thrombolysis, of which 97% decreased or stopped tissue plasminogen activator based on the level. Self-reported estimates of significant bleeding events during thrombolysis were 1.86% in those who monitored fibrinogen and 1.93% in those who did not. Only 34% of all respondents report, in their clinical experience, having found low fibrinogen level to be correlated with bleeding events. There was no significant difference in self-reported major bleeding rates between practitioners who monitor and those who do not monitor fibrinogen. This high variability of clinical use of fibrinogen monitoring during catheter-directed thrombolysis is secondary to the paucity of scientific studies demonstrating its utility; further scientific investigation is needed to define the true utility of fibrinogen monitoring.

## 1. Introduction

Acute thrombosis is a commonly encountered clinical problem associated with high morbidity and mortality [1]. Potential therapies include systemic anticoagulation, catheter-directed thrombolysis (CDT), surgical revascularization, and even limb amputation. The choice of therapy depends on the chronicity of the clot, degree of thrombosis, status of the limb, available resources, and practitioner preferences. After three randomized multicenter trials published in the 1990s showed clinical efficacy and safety of CDT, for selected patients, it became the procedure of choice [2,3,4,5].

Deep venous thrombosis presents in approximately 100 people per 100,000 [6]. It is associated with a high rate of both acute and chronic morbidity. Acutely, patients can present with swelling, pain, or more serious symptoms such as associated pulmonary embolism, phlegmasia cerulean dolens, or venous gangrene. Long-term sequelae can include debilitating post-thrombotic syndrome (PTS) or chronic pain and swelling [7]. While a recently completed multicenter randomized control trial (ATTRACT Trial) failed to show a decrease in the rate of PTS when compared to conservative therapy of anticoagulation and compression stockings, the severity of PTS was decreased in those who underwent CDT [8].

Lower extremity arterial lysis is typically reserved for patients with Rutherford Classification I (viable limb not acutely threatened) and stage IIa (marginally threatened) limbs and has been shown in these select cases to have equal efficacy compared to surgical revascularization [9].

Catheter-directed thrombolysis is not without risk. The literature reports an 8.8% risk of major hemorrhage and a 43.8% risk for minor hemorrhage [10,11]. Patients undergoing catheter-based thrombolysis require overnight admission with a high level of care, often ICU, to monitor for signs of bleeding or complications from therapy. In addition to monitoring for clinical signs of bleeding, laboratory values have been used both to identify occult bleeding (serial hemoglobin and hematocrit) as well as to predict future bleeding.

**Figure 1:**
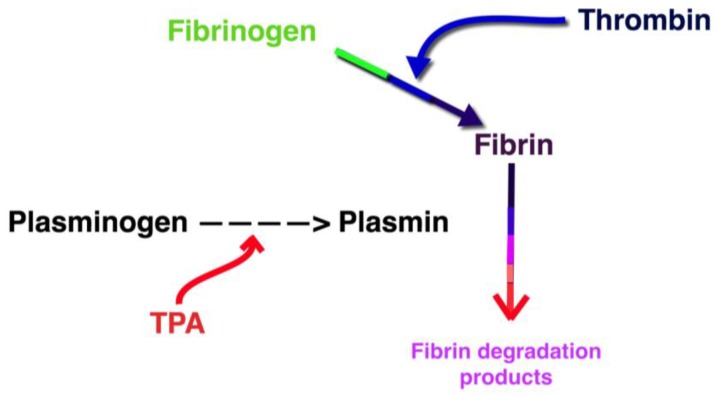
Fibrinogen degradation pathway. TPA: tissue plasminogen activator.

Fibrinogen is a soluble circulating glycoprotein and is converted to fibrin by thrombin as part of the final common pathway in the coagulation cascade. Normal circulating fibrinogen levels range from 200–400 mg/dL with a half-life of 4 days [12]. Plasminogen is converted into plasmin by tissue plasminogen activator (TPA), urokinase, factor XIa, and factor XIIa. Plasmin is a proteolytic enzyme that cleaves the fibrin in thrombi into fibrin degradation products (Figure 1). Fibrinogen levels are used to predict bleeding during CDT and guide TPA dosing. However, low levels of fibrinogen are nonspecific and clinical meanings are controversial. It has been associated with both an increased risk of bleeding as well as prothrombotic conditions [3,13,14,15,16]. The purpose of our study was to investigate the current clinical use of monitoring fibrinogen levels during thrombolysis.

## 2. Materials and Methods

A voluntary and anonymous online survey was sent to all physician members of the Society of Interventional Radiology (*n* = 3890) after approval by the society. The survey consisted of 23 questions related to practitioner demographics, thrombolysis protocol, and fibrinogen monitoring (Appendix A). The survey was available from 10 September 2016 until 20 September 2016. Practice trends were evaluated. When possible, statistics were performed to evaluate the difference in reported outcomes between practitioners who monitored fibrinogen and those that did not using the Student’s *t*-test.

## 3. Results

**Figure 2:**
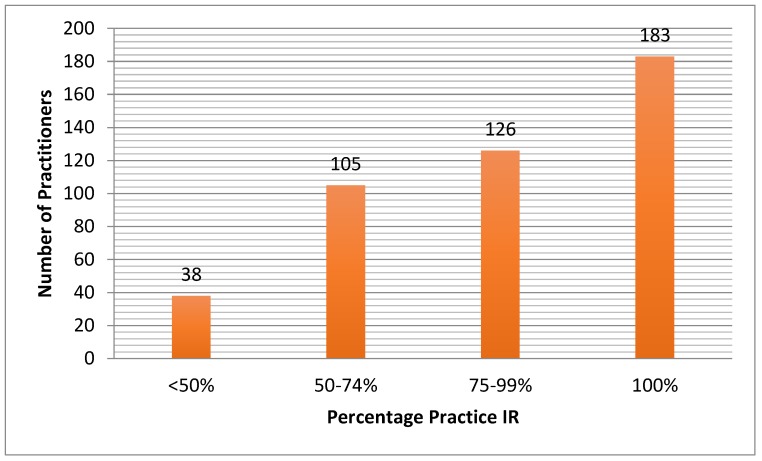
Percentage of practice that is interventional radiology (IR) of the respondents of the survey.

There were 455 practitioner responses (11.7%). Two hundred and eighty-three (62.2%) respondents were in private practice, 156 (34.3%) were in academics, with the remainder in government practices or multispecialty clinics. The average number of years in practice was 13 years, ranging from <1 to 45 years. The majority of practitioners dedicate >75% of their clinical time doing interventional radiology (IR) (Figure 2).

**Figure 3:**
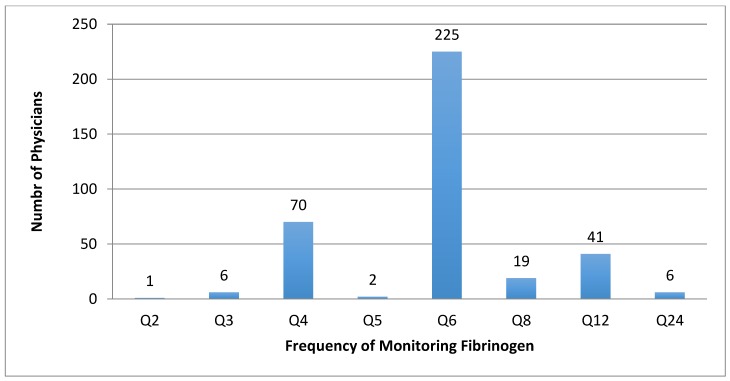
Frequency (in hours) with which plasma fibrinogen levels are monitored in those who routinely monitor during thrombolysis procedures. Q2 = every 2 h, Q3 = every 3 h, Q4 = every 4 h, Q5 = every 5 h, Q6 = every 6 h, Q8 = every 8 h, Q12 = every 12 h, Q24 = every 24 h.

Three hundred and seventy-four respondents (82%; *n* = 81) monitored fibrinogen during thrombolysis, and 18% do not monitor fibrinogen. The most frequent interval for fibrinogen blood draws was every 6 h (*n* = 225; 49.5%), ranging from 2 to 24 h (Figure 3).

**Figure 4:**
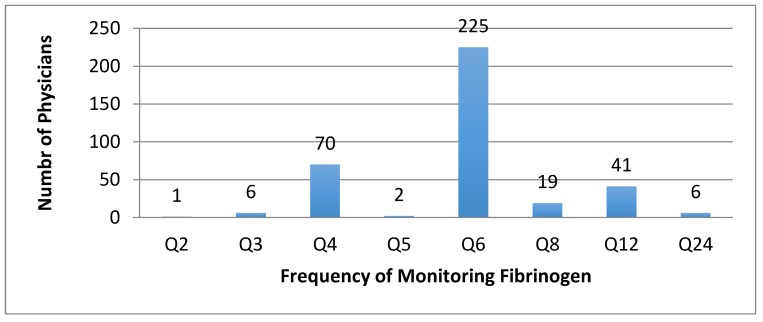
Reported protocol for frequency of monitoring fibrinogen levels during thrombolysis. Q2 = every 2 h, Q3 = every 3 h, Q4 = every 4 h, Q5 = every 5 h, Q6 = every 6 h, Q8 = every 8 h, Q12 = every 12 h, Q24 = every 24 h.

Adjustment of TPA dose based on fibrinogen levels was variable. Of those that monitored fibrinogen, 10 respondents (2.2%) did not decrease the TPA dose based on falling fibrinogen, but would give cryoprecipitate or fresh frozen plasma (FFP). In the remaining respondents who did adjust TPA based on fibrinogen levels (*n* = 364), the threshold fibrinogen level that prompted TPA adjustment varied. The most common level at which TPA was held was at 100 mg/dL (*n* = 143; 31.4%), with a range of 40–150 mg/dL. The most common fibrinogen level at which to decrease the dose of TPA was 150 mg/dL (*n* = 105; 23.1%), with a range of 40–300 mg/dL. Twenty-four practitioners (5.3%) decreased the TPA dose based on a percentage drop from the baseline, rather than an absolute value. Once TPA was held, there was variability in the protocol for rechecking fibrinogen prior to restarting thrombolytics, with most restarting between 2 and 6 h (Figure 4). Three practitioners did not recheck fibrinogen once they stopped thrombolytics, three practitioners performed an angiogram prior to rechecking fibrinogen, and three practitioners would recheck fibrinogen only after giving cryoprecipitate.

**Figure 5:**
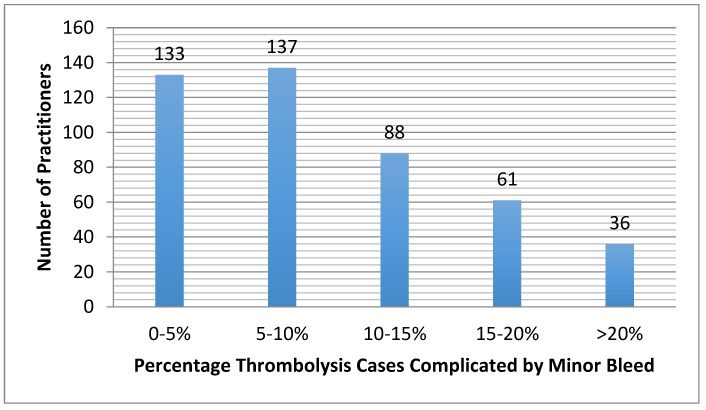
Self-reported percentages of cases that are complicated by minor bleeding, such as oozing at the access site or blood in stool without a significant drop in hematocrit.

**Figure 6:**
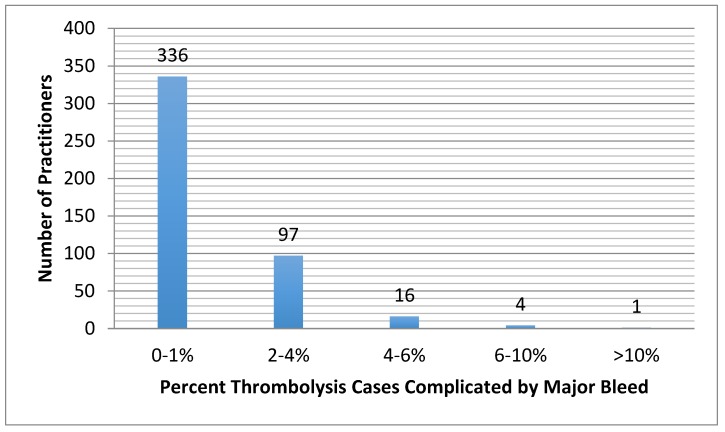
Self-reported percentage of thrombolysis cases that are complicated by major bleeding, including intracranial hemorrhage, gastrointestinal bleed requiring transfusion or intervention, or retroperitoneal bleed requiring transfusion or intervention.

**Figure 7:**
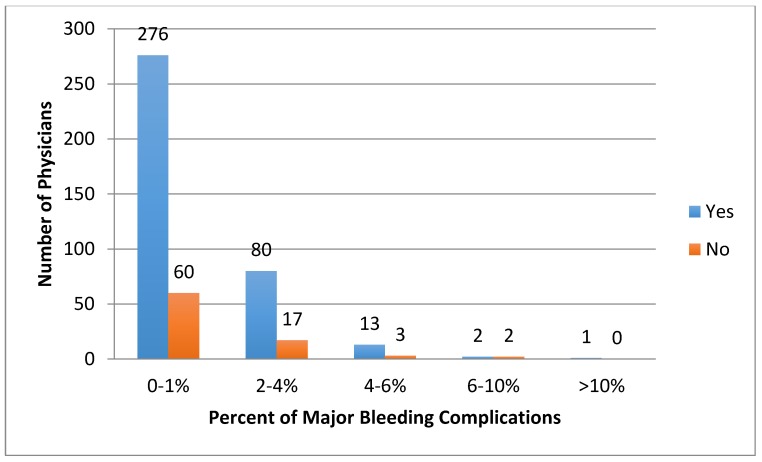
Comparison of self-reported percentages of thrombolysis cases that are complicated by major bleeding complications between practitioners who monitor fibrinogen and those that do not (*p* = 0.15).

Respondents reported the percentage of their thrombolysis cases (both arterial and venous) that were complicated by minor bleeding (e.g., oozing from the access site, or blood in stool without a significant decrease in hematocrit) (Figure 5) and major bleeding (e.g., intracranial hemorrhage gastrointestinal bleed requiring transfusion/intervention, or retroperitoneal bleed requiring transfusion or intervention) (Figure 6). Rates of these events did not significantly differ between those who monitor fibrinogen and those that do not (*p* = 0.15) (Figure 7). When asked if in one’s clinical experience they found that low fibrinogen levels to lead to bleeding, 59% (*n* = 220) responded that they did not find that low fibrinogen clinically correlated with bleeding.

Use of adjunctive systemic anticoagulation during thrombolysis varied. The majority of practitioners (*n* = 338; 76%) utilized systemic anticoagulation. The majority used heparin alone (*n* = 325; 71.4%) (Table 1). The varied modes of administration of systemic anticoagulation comprised via a sheath (*n* = 210), through a peripheral IV (*n* = 100), through the sheath and peripheral IV (*n* = 2), through a peripherally inserted central catheter (PICC; *n* = 5), or varied practice (*n* = 14). For those practitioners who used heparin, the majority used a set dose of 500 units/h (*n* = 94); however, there is a broad range of protocols of heparin use, including those that are weight-based, protocol-driven, or partial thromboplastin time (ptt)-driven (Table 2). The majority of practitioners (*n* = 374; 82.2%) monitor hemoglobin and hematocrit levels.

## 4. Discussion

Both arterial and venous CDT are commonly performed procedures. In addition to the technical aspects, interventional radiologists play a primary role in patient management. While the potential benefits of the procedure are big, including limb- or life-saving, a significant risk of this procedure is bleeding. Fibrinogen is often used as a surrogate to estimate the risk of hemorrhage and guide TPA dosage to avoid bleeding. Currently, there is a paucity of evidence-based literature or consensus guidelines to guide the optimal use of fibrinogen in CDT. 

Previous studies have reached different conclusions regarding the relationship of fibrinogen levels and the risk of bleeding. One study reviewed 69 thrombolysis procedures in patients with acute lower extremity thrombus, with a total of 10 major bleeding events. They showed a significantly higher rate of bleeding if the fibrinogen level was <150 mg/dL [16]. On the contrary, a study performed by Lee et al., which examined 49 procedures, found that there was no significant increased risk of bleeding in patients with fibrinogen levels less than 150 mg/dL, despite larger total doses of TPA and longer duration of therapy [17]. The prospective PURPOSE trial with 241 patients undergoing catheter-directed thrombolysis found that there was no statistically significant difference in major or minor bleeding in patients with fibrinogen >100 mg/dL or <100 mg/dL (*p* = 0.108) [14]. A meta-analysis found that there was wide variety in fibrinogen protocols, drawing the conclusion that the value of plasma fibrinogen to predict hemorrhage in CDT remains unclear [18]. Interestingly, a retrospective review of 32 patients who underwent catheter-directed thrombolysis found a significant correlation with the percentage of decrease of fibrinogen and degree of clot burden resolution, suggesting that falling fibrinogen levels during CDT were associated with efficacy of the procedure and not bleeding risk [19]. 

The lack of a large prospective randomized trial or consistent results in smaller retrospective studies is reflected in a 2013 Society of Interventional Radiology consensus statement on Quality Improvement Guidelines for Percutaneous Management of Acute Lower-Extremity Ischemia from the Society of Interventional Radiology. In this, it states that given the lack of evidence to show that laboratory monitoring of fibrinogen will predict adverse bleeding, they neither recommend nor dissuade its use [20].

Practice patterns found in our survey reflect the inconclusive findings in the literature and the lack of consensus guidelines. This study showed wide variations on whether fibrinogen was monitored at all, what fibrinogen level was considered actionable, and what action was taken. Additionally, our study found no significant difference in self-reported major bleeding events between those who monitored fibrinogen and those who do not. Potential drawbacks of fibrinogen monitoring include the laboratory costs, added complexity in patient management, and potential early termination of/decrease of dose of TPA. 

While the findings of our study are limited due to the study design and recall bias, we feel that this data brings to light the lack of standardization of practice in monitoring of thrombolysis patients. Further prospective studies are needed to evaluate its efficacy to help standardize practice in the hopes of improving patient care and safety.

## 5. Conclusions

There is a paucity of literature to support the common clinical practice of monitoring fibrinogen levels during thrombolysis and its use as a predictor of patient bleeding. Our study highlighted the widely varied clinical practice and lack of standardization not only for whether or not to monitor fibrinogen, but how to respond to different fibrinogen levels. Further research to determine the value of monitoring fibrinogen is needed.

## Figures and Tables

**Table 1:** Type of systemic anticoagulation used during thrombolysis.

Type of Anticoagulation	Number of Practitioners
Heparin alone	325
Arterial heparin for venous enoxaparin	1
Heparin or, if allergy, argatroban	1
Heparin or lovenox	2
Heparin or bivalirudin	1
Heparin or aggranox	1
Heparin or low-molecular-weight heparin	1

**Table 2:** Systemic heparin protocol.

Varied: Unspecified		20
Weight-based protocol		21
Protocol-driven	Low dose	12
	High dose/therapeutic	4
Partial thromboplastin time	40–45	1
	40–50	2
	40–60	9
	45–65	1
	50	1
	50–60	4
	50–65	1
	50–70	2
	50–75	1
	60	2
	>60	1
	60–70	1
	60–80	5
	60–90	1
	70–80	1
	70–90	1
Set dose in units/h	75	1
	100	3
	100–500	1
	200	8
	250	3
	300	29
	200–300	3
	200–400	3
	200–600	1
	250–500	1
	300–500	9
	400	30
	450	1
	400–500	5
	400–600	1
	400–800	2
	400–1500	1
	500	94
	500–600	4
	500–700	2
	500–800	3
	500–1000	2
	600	3
	600–800	1
	800	3
	800–1000	1
	1000	8
	1000–1200	1
	2000	1
	2000–5000	1
	3000	1
	5000	4

## Appendix A

1. What type of institution do you practice? What type of institution do you practice?

Private

Academic

Government

Other (please specify)

2. What percentage of your practice is interventional radiology?

0

100

3. How many years have you been practicing?

4. What percent of your thrombolysis cases have been complicated by a major bleed (includes intracranial hemorrhage, GI bleed or retroperitoneal bleed necessitating transfusion or intervention)?

0–1%

2–4%

4–6%

6–10%

>10%

5. What percent of your thrombolysis cases have been complicated by a minor bleed (includes oozing at the access site, blood in stool without significant decrease in hematocrit)?

0–5%

5–10%

10–15%

15–20%

>20%

6. In your experience have you found that low fibrinogen levels lead to bleeding?

Yes

No

Other (please specify)

7. Do you draw a pre-thrombolysis fibrinogen level?

Yes

No

8. Do you monitor fibrinogen levels during thrombolysis cases (if yes proceed to question 9, if no proceed to question 19)?

Yes

No

If you answered yes to question 8 please proceed with question 9. If you answered no please proceed to question 19

9. How often do you monitor fibrinogen levels?

Q2

Q3

Q4

Q5

Q6

Q12

Other (please specify)

10. Do you change therapy based on the fibrinogen level (if yes proceed to question 11, if no proceed to question 12)?

Yes

No

11. If you answered yes to question 10, how do you change your thrombolysis protocol based on the fibrinogen level?

Stop TPA

Decrease dose of TPA

Other (please specify)

12. At what fibrinogen level do you stop TPA?

13. At what fibrinogen level do you decrease TPA?

14. By how much do you decrease TPA (percent from starting dose)?

15. If you adjust or stop thrombolysis do you recheck fibrinogen level prior to restarting thrombolysis (if yes proceed to question 16, if no proceed to question 17, if N/A proceed to question 18)?

Yes

No

N/A

16. If you answered yes to question 15 when (how many hours after stopping thrombolysis) do you recheck the fibrinogen level?

0–1 h

1–2 h

2–4 h

4–6 h

6–8 h

8–12 h

Other (please specify)

17. If you answered no to question 15, when (how many hours) do you restart thrombolysis?

18. When restarting thrombolysis at what percentage of the original dose do you restart?

19. Do you have systemic anticoagulation running during thrombolysis (if yes proceed to question 20, if no proceed to question 23)?

Yes

No

20. If you answered yes to question 19 what agent is used?

21. If you answered yes to question 19 what dose?

22. If you answered yes to question 19 how is it administered?

Through the sheath

Through a peripheral IV

Subcutaneously

Other (please specify)

23. Do you monitor hematocrit and hemoglobin while doing thrombolysis?

Yes

No

Other (please specify)
